# Effectiveness of biomedical interventions on the chronic stage of traumatic brain injury: a systematic review of randomized controlled trials

**DOI:** 10.3389/fneur.2024.1321239

**Published:** 2024-03-18

**Authors:** Keisuke Kawata, Devin J. Rettke, Christopher Thompson, Rebekah Mannix, Jeffrey J. Bazarian, Dibyadyuti Datta

**Affiliations:** ^1^Department of Kinesiology, Indiana University School of Public Health-Bloomington, Bloomington, IN, United States; ^2^Department of Pediatrics, Indiana University School of Medicine, Indianapolis, IN, United States; ^3^Program in Neuroscience, The College of Arts and Sciences, Indiana University, Bloomington, IN, United States; ^4^Division of Emergency Medicine, Boston Children's Hospital, Boston, MA, United States; ^5^Department of Pediatrics, Harvard Medical School, Boston, MA, United States; ^6^Department of Emergency Medicine, University of Rochester School of Medicine and Dentistry, Rochester, NY, United States

**Keywords:** traumatic brain injury, pharmacology, stimulation, exercise, chronic traumatic encephalopathy

## Abstract

Traumatic brain injury (TBI), in any form and severity, can pose risks for developing chronic symptoms that can profoundly hinder patients’ work/academic, social, and personal lives. In the past 3 decades, a multitude of pharmacological, stimulation, and exercise-based interventions have been proposed to ameliorate symptoms, memory impairment, mental fatigue, and/or sleep disturbances. However, most research is preliminary, thus limited influence on clinical practice. This review aims to systematically appraise the evidence derived from randomized controlled trials (RCT) regarding the effectiveness of pharmacological, stimulation, and exercise-based interventions in treating chronic symptoms due to TBI. Our search results indicate that despite the largest volume of literature, pharmacological interventions, especially using neurostimulant medications to treat physical, cognitive, and mental fatigue, as well as daytime sleepiness, have yielded inconsistent results, such that some studies found improvements in fatigue (e.g., Modafinil, Armodafinil) while others failed to yield the improvements after the intervention. Conversely, brain stimulation techniques (e.g., transcranial magnetic stimulation, blue light therapy) and exercise interventions were effective in ameliorating mental health symptoms and cognition. However, given that most RCTs are equipped with small sample sizes, more high-quality, larger-scale RCTs is needed.

## 1 Introduction

Despite nearly a century of dedicated research, traumatic brain injury (TBI) remains a significant public health concern, contributing to both morbidity and mortality on a global scale (1, 2). It is evident that the sequelae of TBI are long-lasting, with symptoms persisting for months to years in a majority of patients with moderate to severe TBI (2). Up to 30% of mild TBI (mTBI)/concussion patients continue to experience symptoms beyond the first month post-injury (3, 4). The timely and accurate diagnosis of TBI is crucial for a prompt recovery, but the impact of appropriate treatment is arguably even more beneficial. Several consensus-based guidelines have been published to guide acute triage and management of TBI (5, 6), but there is a notable absence of universally accepted treatments for the chronic sequelae of TBI, including concussions.

The quality of life remains a vital attribute of individuals grappling with TBI (7). Beyond the evident physical and cognitive disabilities, TBI patients often suffer from psychiatric disorders, encompassing affective, anxiety, post-traumatic stress disorders (PTSD), and sleep disturbances (8, 9). These psychiatric burdens of TBI are largely invisible, yet studies have begun unraveling potential countermeasures for not only physical and cognitive symptoms but also mental health issues stemming from TBI. However, unfortunately, multidisciplinary neurorehabilitation is often inaccessible to the majority of TBI patients. Over the past 3 decades, a myriad of interventions, including pharmacological, stimulation, and exercise-based approaches, have emerged to ameliorate symptoms, such as mental fatigue, and sleep disturbances. For example, Johanson et al. (10) prescribed a four-week course of the stimulant medication (Methylphenidate) to adults experiencing chronic mental fatigue symptoms after a mild to moderate TBI. The results were promising, as Methylphenidate significantly improved mental fatigue symptoms, such as stress, slowness of thinking, difficulty concentrating, and lack of initiative, in a dose-dependent manner. Additionally, other neurostimulants, such as Modafinil (11), Armodafinil (12), and Monoaminergic (13) have demonstrated efficacy as countermeasures for post-TBI fatigue and persistent sleepiness during the post-acute phase of recovery. In a separate randomized controlled trial (RCT), the administration of doxycycline, an antibiotic medication, during the acute phase in TBI patients resulted in significant reductions in the neural injury blood biomarker (neuron-specific enolase) and improvement in the Glasgow Coma Scale over a one-week treatment period compared to a placebo group (14).

In another scope of research, the use of neural stimulation as a therapeutic strategy for TBI has attracted considerable attention. Among these approaches, transcranial magnetic stimulation (TMS) stands out as one of the most extensively explored techniques, designed to elicit repetitive neural activation on the cortical surface with the goal of restoring neural network functionality. Small-scale RCTs implemented repetitive TMS (rTMS) 5 days a week for 2 weeks in patients with mild to moderate TBI. The results indicated that the rTMS intervention was associated with a reduction in pain score and depression symptoms (15, 16), along with improvements in working memory and processing speed (17, 18). However, divergent outcomes emerge from alternative lines of research where the application of rTMS following mild to moderate TBI showed no discernible positive impact on mental health symptoms and demonstrated negligible effects on cognitive function (19) and reducing headaches (20). These conflicting findings, combined with the exploration of other emerging stimulation approach, such as acupuncture and transcranial direct-current stimulation, underscore the necessity for a systematic evaluation of the current landscape of neurostimulant therapy in TBI outcomes, particularly for patients who are living with lingering physical and psychological symptoms.

A groundbreaking study conducted by Dr. John Leddy and his colleagues (21) has played a pivotal role in revolutionizing concussion management through the implementation of a regulated aerobic exercise protocol (22). The early integration of exercise into the recovery process from TBI is thought to stimulate cerebral blood flow (23), trigger the overexpression of neurotrophic factor (e.g., BDNF) to promote neuronal repair (24, 25), and contribute to the regulation of mental health symptoms (26). In a RCT involving adolescents with concussion, individuals assigned to a 20-min daily exercise protocol exhibited significantly faster recovery and a 48% reduced risk of developing persistent post-concussive symptoms (PPCS) compared to their counterparts following a daily stretch protocol (27). These findings were subsequently validated by a recent RCT conducted among adolescents and young adults with concussions (28). While considerable progress has been made in the past decades, leading to promising interventions for TBI, the majority of systematic evaluations for treatment effectiveness has focused on the acute phases of TBI. This underscores a significant knowledge gap and emphasizes the necessity for a systematic review to explore treatment options specifically tailored for the chronic stage of TBI.

This systematic review aims to determine the effectiveness of pharmacological, stimulation, and exercise interventions in alleviating physical, cognitive, and psychiatric symptoms among adult TBI patients experiencing persistent symptoms lasting at least 1-month post-injury. The outcomes of this review aim to offer empirical evidence for individuals grappling with chronic symptoms stemming from various degrees of TBI.

## 2 Materials and methods

This systematic review was conducted in accordance with Preferred Reporting Items for Systematic Reviews and Meta-Analyses (PRISMA) guidelines (29).

### 2.1 Data sources and search strategy

A systematic review of the current literature was performed by two independent reviewers (DR and CT) using the electronic databases *PubMed, EBSCO*, and *Web of Science*. The search timeframe included studies from 1 January 1990 to 1 September 2020. The following keywords were used in different combinations: traumatic brain injury, TBI, concussion, post-concussion syndrome, persistent post-concussive symptom, post-concussive disorder, treatment, and therapy. For the complete list of combinations, see Table 1. Cited papers within articles meeting the selection criteria were also collected. Searches were limited to RCT, human participants, and English language publications. All records of literature search were examined by title and abstract to exclude irrelevant records. All abstracts that are related to TBI involving treatment or therapeutic interventions were selected for a full reading of the article.

**Table 1:** Summary of search terms.

Search term	Total	Abstracts reviewed	Articles included
Traumatic brain injury, treatment	965	204	35
Traumatic brain injury, therapy	947	0	0
TBI, treatment	593	3	2
TBI, therapy	580	0	0
Concussion, treatment	139	23	8
Concussion, therapy	140	0	0
Post-concussion syndrome, treatment	59	3	0
Post-concussion syndrome, therapy	59	0	0
Persistent post-concussion symptom, treatment	35	0	0
Persistent post-concussion symptom, therapy	35	0	0
Post-concussive disorder, treatment	14	0	0
Post-concussive disorder, therapy	14	0	0

### 2.2 Inclusion and exclusion criteria

This systematic review included RCTs that evaluate the effectiveness and/or efficacy of treatment or therapeutic interventions for TBI. The scope of this review included pharmaceutical, biomedical, and physical aspects of interventions/treatments, whereas vestibulo-ocular rehabilitation was not included in this study because of the recent abundance in available systematic reviews and meta-analysis (30–32). Also, homeopathic medicine was not included in this review, given the ongoing debate surrounding its efficacy and safety. This review focuses on TBI in adult civilian population (>18 years old) because the neurodevelopmental rate varies between pediatric patients, and this neurodevelopmental effect makes an interpretation of therapeutic effectiveness difficult. Furthermore, military TBI are often complicated with emergence of PTSD and other psychiatric issues not originated from brain trauma; thus, this review focused on civilian population. Additional exclusion criteria were foreign language papers other than English, conference abstracts, studies that are not RCT (e.g., cohort studies, case studies, animal studies), editorials, magazine articles, and papers that did not fall within the three main topics. Review articles were considered separately and incorporated into the discussion for context.

After the systematic filtration, papers were categorized into the three main domains, which yielded four subareas of interventions per category.

1. Pharmacological intervention focuses on pharmaceutical treatments that included hormone therapy, anti-inflammatory treatment, and neurotransmitter modulation treatment.
2. Stimulation-based intervention uses electrical- or magnetic-based stimulation techniques to excite or inhibit neural signaling, including stimulation techniques like cutaneous, transcutaneous, optical, and transcranial stimulation.
3. Exercise-based intervention aims to increase blood circulation and promote endogenous healing processes, and the interventions range from independent and dependent activities, virtual reality, to an alteration of body temperature.

### 2.3 Operational definitions

The chronic phase was operationally defined as patients exhibiting persisting signs and symptoms of TBI after 30 days of injury. In this review, the Glasgow Coma Scale (GCS) was used to categorize the initial severity of TBI. The GCS is based on motor responsiveness, verbal performance, and eye opening to appropriate stimuli with scores ranging from 3 being the most severe to 15 being unaffected. A mTBI/concussion is defined by a score between 13 to 15, a moderate TBI from 9 to 12, and a severe TBI from 3 to 8, which represents when the individual is unresponsive. While the majority of papers used GCS to classify the severity of TBI during an admission, this review is focused on papers describing chronic, lingering aspects of TBI at least 30 days from the initial injury. Therefore, the initial GCS score may not accurately reflect the status of patients at the time of intervention. While the Glasgow Outcome Scale (GOS) is the desirable measure in the chronic stage, there was a very large variability between papers in terms of the assessments of neurological status. Regardless of this limitation, the goal of this systematic review was achievable.

### 2.4 Data extraction and quality assessment

Two authors (DR and CT) independently performed the identification, screening, eligibility and inclusion of studies, with disagreement settled by the senior author (KK). The following was recorded: first author, year of publication, study design, age and number of patients, time since injury, GCS score, treatment methodology, main outcome. When included studies refer to previous papers for details of their methods, full texts of these references were screened, and available data was extracted. In addition, data listed in supplementary documents will also be extracted to present a fully comprehensive review of the literature.

### 2.5 Risk of Bias selection

The risk of bias was assessed using the Cochrane Collaboration’s tool for assessing risk of bias (33). The risk of bias was assessed in the context of selection bias (sequence generation and allocation concealment), performance bias (blinding of participants and research personnel), detection bias (blinding of analysis), and reporting bias. The outcomes of the assessment are listed in Supplementary Table 1.

## 3 Results

### 3.1 Search outcome

**Figure 1:**
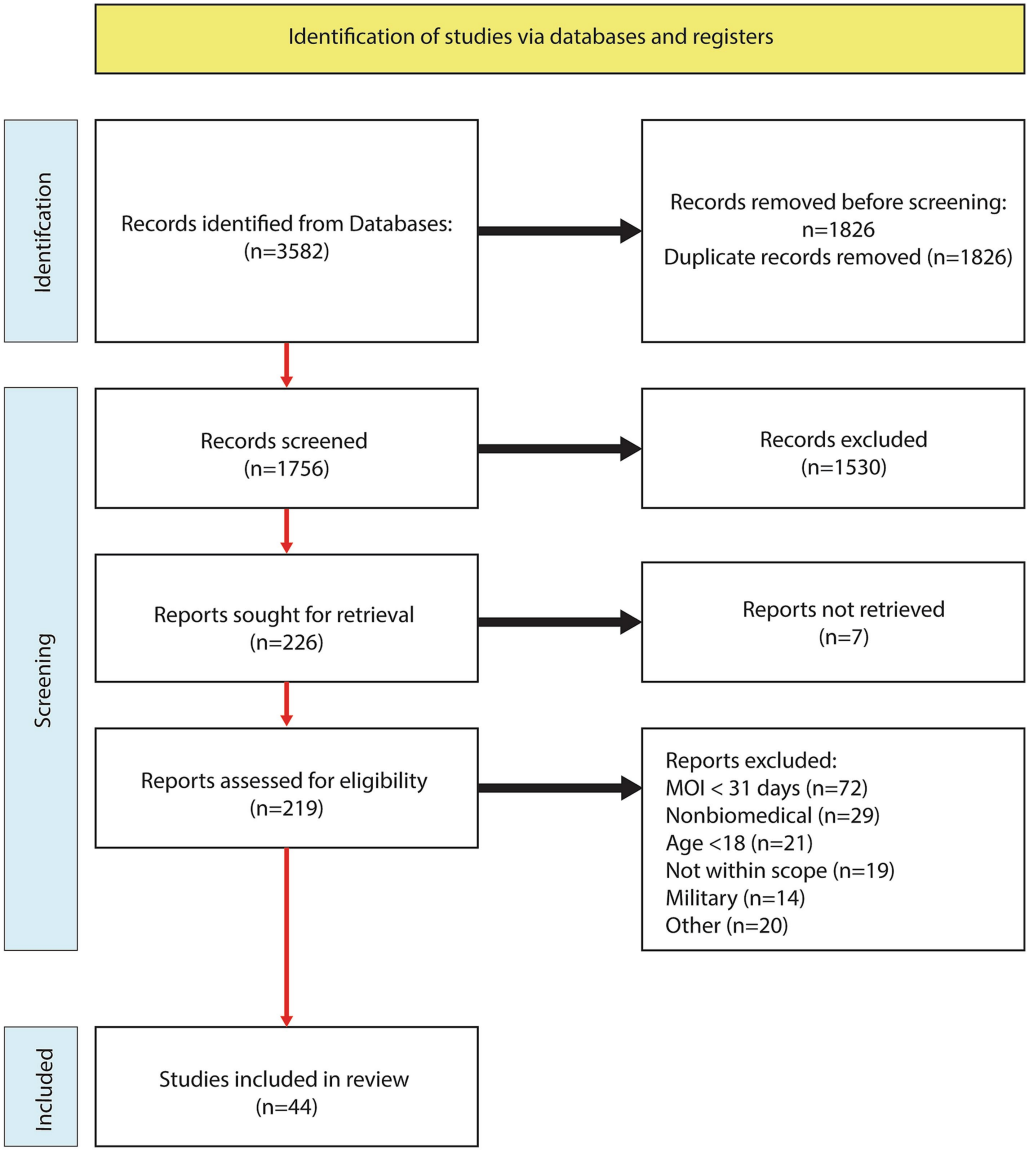
PRISMA flow chart.

The systematic literature search yielded 1,756 abstracts after filtering for duplicates, and 219 abstracts were assessed and advanced to the full-text review. Following the analysis of 219 full text articles, an additional 175 were excluded. Reasons for exclusions were time-since-injury < 30 days (*n* = 72), nonbiomedical (*n* = 29), age < 18 (*n* = 21), not within scope (*n* = 19), military (n = 14), and other (*n* = 19). See the study flow chart for details (Figure 1). A total of 44 articles met the inclusion criteria and further categorized into pharmacological (*n* = 19: Table 2), stimulation (*n* = 14: Table 3), and exercise (*n* = 12: Table 4).

**Table 2:** RCT using pharmacological interventions.

Authors (and year)	Population	Time since injury	Intervention (type, dosage, duration, frequency)	Primary outcome measures	Outcome findings
Tenovuo (2004)	111 adults with TBI	71 ± 6.7 months	Central acetylcholinesterase inhibitor (CAI): 27 patients took donepezil once a day (7.2 mg mean), 30 took galantamine twice a day (5 mg mean), and 54 took rivastigmine twice a day (2.3 mg mean). Average duration of the treatment was 18.4 months (6–33 months).	Patient-Reported Outcomes, Glasgow Outcome Scale—Extended	No improvement in self-reported outcomes and Glasgow outcome scale was detected across all 3 treatment types.
	Mild—58%				
	Moderate—10%				
	Severe—32%				
Zhang (2004)	18 adults with TBI (the severity ranged from GCS 3–15)	Cross-over design	Donepezil: A-B vs. B-A design with Donepezil (A) or placebo (B) for 10 weeks each with a 4-week washout period. Dosage was 5 mg/d for the first 2 weeks then 10 mg/d for the last 8 weeks	Auditory Immediate Index (AII), Visual Immediate Index (VII), Wechsler Memory Scale-III, and Paced Auditory Serial Addition Test (PASAR)	Intragroup comparison showed significant increases in AII, VII, and PASAT scores compared to baseline, whereas placebo condition did not change any of the outcomes.
		Group A: 4.6 ± 0.7 months			
		Group B: 3.9 ± 0.5 months			
Kim (2006)	18 adults with TBI (no description of the initial TBI severity)	Treatment: 1.6 ± 0.5 years	Methylphenidate: 20 mg or placebo was given 2 days after baseline then participants were tested 2 h and 2 days post treatment.	Working memory task and endogenous visuospatial attention tasks	The treatment group showed a significant improvement in working memory tasks and visuospatial attention when compared to placebo at 2 h post, but no group difference was found after 2 days.
		Control: 3.6 ± 3.4 years			
Silver (2006)	157 adults with mild to severe TBI	Con: 103.4 ± 85.1 months	Rivastigmine: Participants would take 3 to 6 mg/day twice a day or a placebo for 12-weeks	Cambridge Neuropsychological Test Automated Battery Rapid Visual Information Processing (CANTAB-RVIP) A’ subtest and the Hopkins Verbal Learning Test (HVLT)	There were no significant differences in CANTAB-RVIP and HVLT scores between treatment and placebo groups.
		Experimental: 79.9 ± 78.7 months			
Amitabh (2008)	51 adults with TBI	Group A: 5.61 ± 4.76 years	Modafinil: Subjects were in a 10-week treatment or placebo phase followed by a 4 week washout period and then switched groups for 10 weeks. The treatment consisted of taking 100 mg/day for 3 days, then 100 mg twice a day for 11 days, followed by 200 mg twice a day for 8 weeks.	Fatigue Severity Scale (FSS), and Epworth Sleepiness Scale (ESS)	No significant differences were found between modafinil and placebo in FSS at week 4 or week 10. Modafnil improved ESS score (less sleepy) at week 4 compared to placebo, but no group difference in ESS at week 10.
	Initial severity:				
	Mild: 25.5%				
	Moderate: 23.5%				
	Severe: 51%	Group B: 5.95 ± 5.29 years			
High (2010)	23 adults with moderate/severe TBI	Con: 5.1 ± 3.6 years	RhGH: Participants took a starting dose of 200 μg/d and increased 200 μg every month up to 600 μg for a year or took a placebo.	Neuropsychological measures: language, visual/spatial functioning, upper extremity motor functioning, information processing efficiency, working memory/attention, learning and memory, executive functioning, intellectual functioning, and emotional functioning	Improvements were found in the treatment group compared to placebo in dominant hand finger tapping test, Wechsler Adult Intelligence Scale III-Information Processing Speed Index, California Verbal Learning Test II, and the Wisconsin Card Sorting Test (executive function)
		Experimental: 11.0 ± 9.2 years			
Kaiser (2010)	20 adults with mild to severe TBI	Treatment: 1.8 ± 0.9 years	Modafinil: Experimental group took 100 mg/day or twice a day if dose was effective and without side effects for 6 weeks, or placebo	Excessive daytime sleepiness (EDS), Epworth Sleepiness Scale, Fatigue Severity Scale	EDS improved significantly when taking Modafinil. There was no difference in posttraumatic fatigue between groups.
		Control: 2.0 ± 1.2 years			
Giacino (2012)	184 adults with severe TBI	Experimental: 36–66 days	Amantadine: 100 mg doses were provided twice a day for 2 weeks, doses then increased to 150 mg for week 3, and 200 mg for week 4 if DRS scores did not improve by 2 points in the experimental group. The control group did not receive Amantadine treatment.	Disability Rating Scale (DRS)	Recovery was significantly faster in the treatment group during the 4-week treatment period. However, after the 2-week washout period, the DRS scores became similar between groups.
		Con: 37–65 days			
Johansson (2013)	24 adults with mild/moderate TBI	Patients: 8.6 ± 0.5.1 years	Methylphenidate: Participants underwent three 4-week trials in one of three sequences: (1) no medication, low dose, normal dose, (2) low dose, normal dose, no medication, or (3) normal dose, no medication, low dose.	Mental Fatigue Scale (MFS)	Normal dose of Methylphenidate led to the greatest improvement in MFS score compared to low and no dose. Low dose also showed modest, but significant improvement in MFS compared to the no dose condition.
Theadom (2013)	60 adults with mTBI	Group A: 7.1 ± 2.66 months Group B: 8.04 ± 2.46 months	Enzogenol: 1000 mg of Enzogenol or placebo for 6 weeks, then Enzogenol for 6 additional weeks for all participants followed by placebo for 4 weeks	Cognitive Failures Questionnaire (CFQ), digit span subtest of the Wechsler Adult Intelligence Scale-III, Arithmetic and Letter Number Sequencing of the Wechsler Adult Intelligence Scale-IV	The treatment condition showed a significant reduction (improvement) in self-reported cognitive failures of the CFQ at week 6 compared to the placebo condition. No other outcomes showed significant result.
Menn (2014)	117 adults with severe TBI	1–10 years	Armodafinil: Participants received 50, 150, or 250 mg/day or a placebo dose for 12 weeks.	Multiple Sleep Latency Test (MSLT), Epworth Sleepiness Scale (ESS), Clinical Global Impression-Change (CGI-C) TBI-Work Instability Scale (TBI-WIS), Clinical Global Impression-Severity Illness (CGI-S), and tolerability	Mean sleep latency was significantly improved in the group taking 250 mg/day compared to placebo. ESS and TBI-WIS scores did not show any significant differences between any group comparison.
Ripley (2014)	55 adults with moderate/severe TBI	Experimental: 8.2 ± 6.1 years	Atomoxetine: Experimental group took 40 mg twice a day for 2 weeks compared to a placebo pill for the control group.	Cognitive Drug Research (CDR) Computerized Cognitive Assessment System	There were no significant group differences in any of the cognitive metrics.
		Control: 6.6 ± 5.5 years			
Hammond (2015)	168 adults with moderate/severe TBI	>6 months	Amantadine: 100 mg doses were given twice a day for 60 days for the treatment group, and an equivalent placebo was given to the control group.	Neuropsychiatric Inventory (NPI-I) Most Problematic Item—a scale for irritability assessment	There were no significant improvements seen in either group for NPI-I Most Problematic item.
Lequerica (2015)	13 adults with mild/moderate TBI	62.1 ± 91.5 months	Ramelteon: nightly dosage of 8 mg or a placebo for a 3-week period followed by a 2 week washout and underwent alternative intervention for 3 weeks.	Sleep/wake patterns, mood, daytime sleepiness, fatigue, and Neurocognitive Index	Total sleep time and executive functioning had a significant increase in the treatment group compared to placebo.
Berginstrom (2017)	64 adults with mild/moderate TBI	Treatment: 8.58 ± 6.84 years	Monoaminergic: Experimental group received 5 mg of -OSU6162 twice a day in week 1, 10 mg twice a day in week 2, 15 mg twice a day in week 3 and 4 compared to placebo group.	Fatigue Severity Scale, Mental Fatigue Scale	Both groups showed significant improvements in both outcomes at follow-up and reported no between group differences.
		Control: 8.10 ± 7.43 years			
Hart (2017)	32 adults with moderate/severe TBI	Experimental: 53.6 ± 25.1 days	Dextroamphetamine (DEX): 10 mg or placebo daily for 3 weeks	Rate of functional recovery, attention, engagement in therapy, and mood	DEX did not lead to significant improvement in any functional and cognitive outcomes.
		Control: 60.2 ± 37.4 days			
Dorer (2018)	14 adults with moderate/severe TBI	23.43 ± 12.25 months	Methylphenidate: Participants took 30 mg or a placebo 75 min prior to an MRI in counterbalanced order. Scans were on average 2 weeks apart.	Sequential finger opposition fMRI paradigm	Methylphenidate resulted in faster reaction times in patients but was not significant compared to controls. fMFI also found the left inferior frontal gyrus to be activated significantly more compared to when on the placebo.
Theadom (2018)	78 adults with mild/moderate TBI	Treatment: 98 days	NeuroAiD II: Participants took MLC901 0.8 g capsules 3 times a day for 6 months or took a placebo.	Cognitive function assessed by the CNS Vital Signs online neuropsychological test.	The treatment group showed significant improvements in executive functioning and complex attention at 6 months compared to the placebo group.
		Control: 94.5 days			

**Table 3:** RCT using brain stimulation interventions.

Authors (and year)	Population	Time since injury	Intervention (type, dosage, duration, frequency)	Primary outcome measures	Outcome findings
Schoenberger (2001)	12 adults with mild to moderate TBI	7.71 ± 5.47 years	Flexyx Neurotherapy System: 25 sessions over 5–8 weeks with 6 min of stimulation via LED strobe frequencies in the range of +5 to +20 Hz to a max of 30 Hz, or control condition without Flexyx treatment.	Individualized Symptom Rating Scale, Beck Depression Inventory (BDI), Multidimensional Fatigue Inventory,	Following treatment, participants had an improvement in reports of depression, fatigue, and other symptoms related to TBI.
McFadden (2011)	42 adults with mild/moderate TBI	Experimental: 23.07 ± 3.41 months	Acupressure: Both groups received eight treatments over 8 weeks. The treatments last 40 min. The control group received treatment in placebo acupoints.	Cognitive impairment and state of being through event-related potentials (ERPs) during Stroop and auditory oddball tasks	Treatment group showed a significant difference compared to the placebo group by having a larger reduction (improvement) in P300 latency and amplitude, as well as a reduced Stroop interference effect. The treatment group showed significantly better performance in working memory task – the digit span, compared to the control group.
		Control: 25.83 ± 4.24 months			
Kang (2012)	9 adults with TBI (no initial severity was specified)	216.9 ± 52.5 days	tDCS: (cross-over design) Experimental: transcranial direct current stimulation of 2 mA for 20 min was applied to the left dorsolateral prefrontal cortex. Sham: a dose of 2 mA for 1 min. Participants were tested directly after intervention, 3 h post, and 24 h post.	Level of attention, fatigue, task difficulty, and sleep quality using a numeric scale from 0 to 10.	No statistically significant group differences were found in rating for attention, fatigue, task difficulty, and sleep quality at any post-intervention timepoint.
Zollman (2012)	24 adults with TBI (no description of the initial TBI severity)	Experimental: 2.17 ± 1.27 years	Acupuncture: Both experimental and control groups met twice a week for 5 weeks with each meeting last 20 min. Experimental group received acupuncture in locations relative for management of insomnia.	Insomnia Severity Index (ISI), Actigraphy, Hamilton Depression Rating Scale (HDRS), Repeatable Battery for the Assessment of Neuropsychological Status (RBANS), and Paced Auditory Serial Addition Test (PASAT)	There was no difference in sleep time between groups but there was a significant improvement in cognitive functioning tested by RBANS and PASAT in the experimental group, whereas no improvement in the control group.
		Control: 3.00 ± 1.85 years			
Lesniak (2014)	23 adults with severe TBI	18.1 ± 19.2 months	tDCS: Experimental participants received a tDCS of 1 mA for 10 min to the left dorsolateral prefrontal cortex followed by cognitive training for 15 consecutive days. Control participants received a tDCS for 25 s followed by the same cognitive training for 15 days.	Battery of memory and attention tests	Test scores showed no group differences 3 weeks prior to and immediately before treatment. Following tDCS treatment, the experimental group showed no significant improvements in cognitive outcome measures compared to the controls.
Sinclair (2014)	30 adults with self-reported TBI (no description of the severity)	Overall sample: 96 days to 13.35 years (1,106 ± 993 days)	Blue Light: Experimental participants underwent either blue light therapy of 465 nm or yellow light therapy of 574 nm pulses compared to the control group who received no treatment. The treatments lasted for 45 min a day for 4 weeks.	Fatigue Severity Scale (FSS) and Epworth Sleepiness Scale (ESS)	The blue light group showed improvements in fatigue compared to yellow light and placebo. Daytime sleepiness was significantly reduced by the blue light treatment. Self-reported depression using ESS reported no significance.
Choi (2018)	12 adults with mild TBI	Sham: 14.3 ± 7.2 months	rTMS: Experimental group underwent 10 sessions (5x a week for 2 weeks) of TMS at 10 Hz with an intensity of 90% of motor threshold and duration of 5-s, for a total of 20 trains separated by 55-s intertrain pauses to the abductor pollicis brevis muscle area of the precentral gyrus on the affected side. The sham group underwent the same stimulation, but the coil was held perpendicular to the skull, preventing any stimulation.	Therapeutic effects and clinical outcomes including central pain which used the numeric rating scale	The rTMS group showed significantly lower pain scale compared to the sham group after 2 week intervention.
		Experimental: 17.0 ± 7.5 months			
Lee (2018)	13 adults with mild/moderate TBI	Sham: 3.88 ± 1.94 months	rTMS: Each session for the experimental group consisted of 50 trains of 40 pulses on each train separated by 25-s pauses applied at 1 Hz to the right dorsolateral prefrontal cortex. Sham group underwent same protocol without any stimulation to the brain. Participants underwent 30-min sessions 5 days a week for 2 weeks.	Depression using Montgomery-Asberg Depression Rating Scale (MADRS) and cognitive function using the Trail Making Test (TMT) and the Stroop Color Word Test (SCWT)	The experimental group showed significant improvement in MADRS, TMT, and SCWT measures after 2-week intervention. While no group difference in all measures at baseline, the experimental group showed significantly better scores in MADRS, TMT, and SCWT compared to the sham group.
		Experimental: 3.85 ± 1.67 months			
Hoy (2019)	21 adults with mild-to-severe TBI	Con:22.11 ± 13.59 years	rTMS: Experimental group were induced repetitive TMS stimulation to the right and left dorsolateral prefrontal cortex. Right sided treatment included a single train of 1 Hz at 110% of resting motor threshold where left sided treatment was 30 × 5-s trains of 10 HZ with 25-s train intervals at 110% of resting motor threshold. Treatment last for 4 weeks for a total of 20 sessions. Sham group underwent the same stimulation but the TMS coil was held away from skull to prevent stimulation.	Montgomery Asberg Depression Rating Scale (MADRS), Inventory of Depressive Symptomatology-Clinician Rated version (IDS-CR) and Self-Rated version (IDS-SR)	rTMS group showed a significant improvement in working memory and executive function compared to sham group. All participants showed improvements in depressive symptoms regardless of experimental or sham group.
		Experimental: 14.73 ± 10.83 years			
Moussavi (2019)	18 adults with mild TBI	Con: 1.6 ± 1.2	rTMS: A total of 13 treatment session over the course of 3 weeks were delivered at a frequency of 20 Hz in trains of 30 pulses with an interval of 10 s at 100% of resting motor threshold to the left dorsolateral prefrontal cortex. A total of 25 trains were given at each treatment. Sham group underwent the same treatment length but with no stimulus to the brain.	Rivermead Post-Concussion Symptoms Questionnaire (RPQ3 and RPQ13)	Significant differences were found using the RPQ13 in the group with injuries occurring less than 12 months prior compared to the sham group.
		Experimental: 1.8 ± 1.4			
Neville (2019)	30 adults with severe TBI	Experimental: 17.62 months	rTMS: Experimental: Stimulation was applied for 10 consecutive days to the left dorsolateral prefrontal cortex at 10 Hz for 5-s and separated by 25-s of no stimulus for a total of 2,000 pulses each day (50 stimuli/train, 40 trains). The sham group underwent the same protocol but with a sham coil producing no stimulus.	Change in executive function using the Trail Making Test (TMT) Part B	There was no difference in TMT Part B test from baseline to after the 10^th^ day or at 90-day follow-up timepoint between groups.
		Con: 18.3 months			
Siddiqi (2019)	14 adults with mild/moderate TBI	Con:8.1 ± 11.3 years	rTMS: Experimental: Treatment consisted of 20 daily sessions of bilateral rTMS using high-frequency left-sided stimulation of 4,000 pulses at 10 Hz with 5-s trainings and 20-s inter-train intervals followed by a low frequency right sided stimulation of a single train of 1,000 pulses with 1 Hz frequency. Sham treatment consisted of using a coil producing very weak pulses not strong enough to cause stimulation.	Montgomery-Asberg Depression Rating Scale (MADRS), temperament and character inventory (TCI), NIH Toolbox Cognitive Battery, Headache Impact Test	There was no improvement in MADRS, TCI, cognitive battery or headache symptoms in the experimental group after the intervention. No group difference was found.
		Experimental: 8.4 ± 8.2 years			
Zhang (2019)	86 adults with TBI (no initial severity was specified)	Treatment: 8.9 ± 2.4 months	NMES: 50 Hz frequency, 250 ms pulse duration, 10 s on and 30 s off, 30 min once a week for 8 weeks or placebo	Post voiding residual urine volume	There were no significant differences between the treatment and placebo groups.
		Control: 9.1 ± 2.7 months			
Killgore (2020)	32 adults with mTBI	Blue light: 6.8 ± 4.4 months	Blue Light: Experimental participants underwent blue light pulses at 469 nm compared to the placebo group of 578 nm of amber light pulses. Both groups treatment was 30 min a day for 6 weeks.	Actigraphically measured sleep and circadian phase shift, subjective sleepiness, and objective sleepiness. Cognitive function was evaluated by self-evaluated questionnaires and Tower of London (TOL).	Blue light reduced daytime sleepiness and improved executive functioning compared to Amber light treatment.
		Amber light: 6.7 ± 3.6 months			

**Table 4:** RCT using exercise-based interventions.

Authors (and year)	Population	Time since injury	Intervention (type, dosage, duration, frequency)	Primary outcome measures	Outcome findings
Jensen (1990)	19 adults with post-traumatic headache	>1 year	Cold Pack: Participants received either manual therapy or cold pack therapy which consisted of 2 rounds of cervical and thoracic mobilization or 2 treatments of a − 14° C cold pack to the neck for 15–20 min. The study lasted 12 weeks.	Pain index for post-traumatic headache	The pain index of the mobilization group at week 5 was significantly lower when compared to the groups using cold packs, as well as pretreatment baseline levels. By week 12 the difference was no longer significant.
Gemmell (2006)	18 adults with mild-to-severe TBI	8.7 years (no SD or range was given)	Tai Chi: Participants attended a 45-min class twice a week for 6 weeks. Controls were placed on a wait-list for a Tai Chi course.	Medical outcome scale short form 36 (MOS SF-36), Rosenberg self-esteem scale (RSES), and Visual analog scales (VAMS)	Tai Chi led to significant improvements on all VAMS scores on 7 mood states (e.g., sadness, energetic, tense) after 6 weeks, as well as between group difference in VAMS. There were no significant differences between groups on the MOS SF-36 or RSES.
Wilson (2006)	38 adults with gait problems due to TBI (No description of the initial TBI severity)	Experimental: 4.0 ± 3.50 months	Ambulation: Both experimental and control group received physical therapy twice daily for 8 weeks. The experimental group additionally performed 1 h of partial weight baring training twice a week.	Functional Independent Measure (FIM), Functional Assessment Measure (FAM), Standing Balance Scale (SBC), Functional Ambulation Category (FAC), Rivermead Mobility Index (RMI), and Gross Motor Subscale (GMS)	Significant improvements were found in FIM, SBC, FAC, and RMI in both the treatment and control group, with no group difference after 8 weeks.
		Control: 2.8 ± 1.80 months			
Blake (2009)	20 adults with mild-to-severe TBI	Con: 14.89 ± 13.62 years	Tai Chi: Intervention participants attended a Qigong exercise class for 1 h per week for 8 weeks. Control participants engaged in non-exercise based social and leisure activities.	General Health Questionnair-12, Physical Self-Description Questionnaire, Social Support for Exercise Habits Scale	Improvements in mood and self-esteem were found after the intervention in the exercise group compared to control at 8-week timepoint.
		Exercise: 16.4 ± 9.04 years			
Driver (2009)	16 adults with TBI	Treatment: 40.75 ± 14.67 months	Exercise: Experimental group had 1-h aquatic program 3 times a week for 8 weeks compared to controls 1-h vocational training 3 times a week for 8 weeks.	Profile of Mood States (POMS)	Significant improvements were found between the treatment and control groups for tension, depression, anger, fatigue, vigor, confusion, and friendliness.
	Self-reported TBI (no description of the severity)	Control: 36.25 ± 14.17 months			
Hoffman (2010)	76 adults with a history of severe TBI	6 months–5 years	Exercise: supervised 30-min aerobic exercise once a week and unsupervised 30-min aerobic exercise 4 times a week for 10 weeks (treatment) or control	Beck Depression Inventory (BDI)	No significant between-group differences in BDI were found at 10 weeks
Wise (2012)	40 adults with TBI	Total: 2.1 ± 1.4 years	Exercise: Treatment condition (supervised 60-min exercise sessions x1/week, plus unsupervised 30-min aerobic exercises x4/week for 10 weeks).	Beck Depression Inventory (BDI), Medical Outcomes Study 12-Item Short-Form Health Survey (SF-12), Perceived Quality of Life Scale	Exercise treatment led to significantly decreased BDI (improved) from baseline to 10 weeks and from baseline to 6 month-follow-up compared to the control condition. SF-12 mental scores improved significantly from baseline to 10 weeks and baseline to 6 months compared to the control condition.
	Self-reported TBI (no description of the severity)		Control condition with no prescribed exercise session.		
Jacoby (2013)	12 adults with moderate to severe TBI	Experimental: 126 ± 85.33 days	Virtual Reality: Experimental participants received ten 45-min Virtual Reality (VR) treatments 3-4x per week. Total duration of the intervention varied between participants. The control participants received 10 sessions of occupational therapy.	Multiple Errands Tests-Simplified Version (MET_SV) and the Executive Function Performance Test (EEPT)	No improvement was shown in MET-SV and EEPT in either group.
		Con: 100 ± 16.76 days			
Bellon (2014)	69 adults with TBI	100.5 ± 119.9 months	Cross-over design: Walking: 12-week walking program with a pedometer, weekly goals, and an assigned coach. Control condition includes only a nutritional control coach call without walking program.	Perceived Stress Scale (PSS) and Center for Epidemiological Studies-Depression (CES-D)	The 12-week walking intervention led to significant improvements in PSS and CES-D, and such improvements were significantly greater compared to a nutrition-based control group.
	Initial severity:				
	Mild: 15%				
	Moderate: 15%				
	Severe: 52%				
	Unknown: 19%				
Kolakowsky-Hayner (2016)	123 adults with TBI (no initial severity was specified)	97.6 ± 113 months	Walking: 12-week walking program with a pedometer, weekly goals, and an assigned coach. Control condition includes only a nutritional control coach call without walking program.	Global Fatigue Index (GFI), Barrow Neurological Institute (BNI) Fatigue Scale Overall Severity Index Score, Multidimensional Fatigue Index (MFI)	No significant improvement was found in GFI, BNI Total and MFI scores after 12-week walking program.
Chiu (2017)	23 adults with TBI	27.6 ± 34.8 months	Warm Footbath: In a crossover study, participants were asked to soak their feet in 41°C water for 30 min for 3 consecutive days or continue with usual care (control).	Sleep efficiency (SE), sleep onset latency (SOL), total sleep time (TST), and wake after sleep onset (WASO)	Warm footbath had a significant reduction in SOL and suppressed WASO. There was no difference in sleep efficiency and total sleep time.
	Initial severity:				
	Mild: 78.3%				
	Moderate: 13%				
	Severe: 4.3%				
Tefertiller (2019)	63 adults with moderate/severe TBI	VR: 8.3 ± 9.2 years	Virtual Reality: Experimental participants performed balance exercises 3-4x a week for 30 min for a total of 12-weeks while receiving feedback through VR system (Xbox Kinect). Control participants performed the same balance exercise without VR system.	Community Balance and Mobility Scale (CB&M)	No significant differences were found between VR and Home Exercise Program in CB&M.
		HEP: 8.5 ± 7.3 years			

### 3.2 Pharmacological interventions

Nineteen studies were examined involving a pharmacological approach (Table 2). Of those, neurotransmitter-modulation treatments (*n* = 14) were the most abundant intervention, followed by anti-inflammatory/oxidant (*n* = 2), and hormone therapy (*n* = 2). The main outcome variable was a cognitive function for 10 papers, including working memory, auditory/visual memory, language, and processing speed, whereas the rest of the papers focused on sleep, physical/mental fatigue, or quality of life. Some of the notable findings are that hormone therapy, such as growth hormone (34) and melatonin (Ramelteon) (35), yielded significant improvement in verbal memory, processing speed, psychomotor functioning, and total sleep duration. Similar improvement in cognitive function was observed in adults with mild to moderate TBI after a 6-month treatment with NeuroAiD II, which has been shown to promote neurite repair and outgrowth (36). Methylphenidate, which is a stimulant medicine typically used to treat attention deficit hyperactivity disorder, has been used in three RCTs (10, 37, 38). Four-week treatment with Methylphenidate was effective in treating mild to moderate TBI patients who suffered from mental fatigue syndrome (10), whereas acute treatment (1 to 2 days) resulted in transient improvements in working memory, attention, and reaction time, yet the beneficial effects disappeared after 2 days (37, 38). Furthermore, fatigue and daytime sleepiness are common lingering symptoms after TBI, and partial improvements in these symptoms were observed in all levels of TBI after several weeks of treatment with Modafinil (neurostimlant) (11), Armodafinil (neurostimlant) (12), or Monoaminergic (neurotransmitter-inducing medicine) (13). Alternatively, doses of Dextroamphetamine (neurostimlant) (39), Atomoxetine (neurostimlant) (40), or Rivastigmine (neurotransmitter-modulating medicine) (41) did not result in improvements in cognitive function or sleep.

### 3.3 Stimulation-based interventions

The current review yielded 14 articles focusing on stimulation interventions (Table 3), with TMS as the most popular approach (*n* = 8), followed by transcutaneous and cutaneous stimulations (*n* = 4) and optical stimulation (*n* = 2). As with the pharmacological interventions, all studies set outcome measures on cognitive function, sleep, or mental symptoms including fatigue, except for one study examining electrical muscle stimulation effects on urine retention post-TBI. rTMS intervention demonstrated mixed results. Two studies demonstrated improved outcomes after a 2-week treatment of rTMS, with significantly improved depression symptoms, working memory, processing speed (17) as well as decreased headache burden (15) in patients with mild to moderate TBI. Others demonstrated no discernible positive impact on mental health symptoms and demonstrated negligible effects on cognitive function (19) reducing headaches (20). Similarly, transcranial direct-current stimulation (tDCS), despite receiving consecutive treatments for 15 days, did not result in any improvements in cognition, sleep, and fatigue (42, 43).

Optical stimulation therapy, in a form of a 4-to-6 week blue light therapy, has produced significantly reduced daytime sleepiness, fatigue, and depression symptoms compared to controls undergoing yellow or amber light therapy (44, 45). Another type of stimulus, acupressure and acupuncture, for durations of 5 to 8 weeks produced significant improvements in cognitive function, including working memory, comprehension skills, and processing speed, but not sleep duration.

### 3.4 Exercise interventions

Twelve RCTs using exercise-based therapy investigated the effectiveness of various interventions that aim to promote the healing process by increasing circulation and changes in body temperature (Table 4). There were independent and dependent activity programs (*n* = 8), virtual reality-based activity (*n* = 2), and temperature therapy (*n* = 2). Patients with all severities of TBI and having lingering symptoms for many years benefited from Tai Chi, where 6 to 8 weeks of Tai Chi lessons significantly improved all domains of mood states, such as sadness, energy, happiness, and tension (46, 47). Exercise classes that lasted at least 1 h 3 times a week for 6 to 8 weeks resulted in improvements in symptoms, such as depression, anger, fatigue, vigor, and friendliness in patients with mild to moderate TBI (48, 49). Conversely, a 30-min aerobic exercise for 10 weeks did not alter mental health symptoms in patients with a history of severe TBI. Interventions such as cold-pack therapy, virtual reality, and walking did not improve cognitive function, sleep, or mental health symptoms (50–52).

## 4 Discussion

Over the past several decades, a vast array of treatments for chronic TBI symptoms have been explored. This systematic review took a novel approach and aimed to evaluate the effectiveness of pharmacological, brain stimulation, and activity-based interventions in the chronic phase (>1-month post) of TBI. There are 4 main findings. First, while many RCTs have used neurostimulant medications to treat physical, cognitive, and mental fatigue, as well as daytime sleepiness, inconsistent results were noted, such that some studies found improvements in fatigue (e.g., Modafinil, Armodafinil) while others failed to realize the improvements after the intervention. Second, in terms of brain stimulation techniques, rTMS showed effectiveness in improving cognitive function and mental health symptoms. Third, blue light therapy induced significant improvements in fatigue and daytime sleepiness in patients with mTBI. Lastly, RCTs outside our scope (e.g., papers published after the search cutoff, 1 September 2020, pediatric TBI) have demonstrated that exercise interventions (e.g., BCTT) may be effective in expediting recovery speed (53–55), especially when an intervention is introduced early on (55). However, RCTs included in this review and using aerobic exercise intervention focused on patients with a history of moderate to severe TBI and indicate that aerobic exercise may not have a strong effect, especially for those who have had a TBI many years ago. Conversely, group exercise like Tai Chi has been shown to improve mental health symptoms.

### 4.1 Pharmacological interventions

The exploration and characterization of pharmacological interventions have remained a dynamic focus in TBI research since the early 2000s, yielding several medications that effectively enhance cognitive functioning and alleviate fatigue/sleepiness post-TBI. Two articles utilized antioxidative treatments consisting of the administration of MLC901 (NeuroAiD II) (36) and Enzogenol (56). MLC901, rooted in traditional Chinese medicine, exhibited the ability to inhibit cerebral inflammation (57) and promote axonal/dendritic healing and neurogenesis following a TBI (58). Enzogenol is a pine bark extract with potent modulatory factors against neurodegenerative cell signaling (59). Although MLC901 and Enzogenol have not been tested in severe TBI, RCTs in mTBI patients were able to detect a significant improvement in executive function and complex attention skills, suggesting that these antioxidative compounds may contribute to neuronal cellular healing processes at least in the milder spectrum of injury. Another pharmacological intervention with positive effects on cognitive functioning and sleepiness is Ramelteon (35). Ramelteon is an FDA-approved melatonin agonist medication to treat patients with insomnia and has also been used to explore the effects after a TBI. This drug has an affinity to two different G-protein-coupled receptors, MT_1_ and MT_2_ in the suprachiasmatic nucleus of the hypothalamus, and regulates the circadian rhythm (60). Following a 3-week nightly dosage of Ramelteon, patients with mild to moderate TBI improved their total sleep time and demonstrated improvements in memory function, psychomotor speed, and cognitive flexibility (35). Of note, in spite of the randomized crossover design, a sample size of 13 patients is underpowered to account for appropriate confounders and potential modulatory factors, warranting a follow-up study with a larger sample size to realize the true effectiveness in post-TBI care.

There were several pharmacological interventions with no promising results, such as Rivastigmine (41) and Atomoxetine (40). Rivastigmine is a cholinesterase inhibitor used to promote mental functioning, including memory and comprehension, and has been used as a treatment for neurodegenerative diseases like Alzheimer’s disease (AD). Silver et al. (41) recruited patients with all severities of TBI in the trial; however, 12 weeks of treatment with Rivastigmine did not improve any aspects of cognitive function compared to a placebo group. This discrepancy between AD and TBI patients is likely due to the level of acetylcholine, such that AD patients often have low acetylcholine levels and benefit from Rivastigmine, whereas acetylcholine levels may not be as depleted after TBI as neurodegenerative conditions. As a result, the RCT by Silver et al. failed to realize the effects of Rivastigmine (41). Atomoxetine is a norepinephrine reuptake inhibitor, often used in patients with attention-deficit hyperactivity disorder (ADHD). This medication is used to increase the levels of norepinephrine in the brain by inhibiting its reuptake into neurons. The abundance of norepinephrine is thought to improve the neural signaling to assist in attention, concentration, and impulse control, which are experienced in some TBI patients. Ripley et al. did not observe Atomoxetine’s positive effects on cognitive function, perhaps due to either the study was conducted in patients who experienced TBI on average 8.2 years ago or not all TBI patients having chief complaint of inattention and impulse control issues.

### 4.2 Brain stimulation

Brain stimulation techniques have evolved in the past decade, allowing researchers and clinicians to administer magnetic stimulations (rTMS) to elicit neuronal signaling. There is a sound theoretical basis and clinical utility in treating various neurological conditions, including chronic pain and rehabilitation for stroke and movement disorders (61). However, this systematic review revealed unique patterns and effectiveness of rTMS in TBI care. All but one studies using rTMS set the dorsolateral prefrontal cortex (DLPFC) as their target region of interest, and seemingly sooner the rTMS intervention was conducted post-TBI (regardless of the severity of TBI), the greater the benefit may be for cognitive function (17), chronic headaches (15), and overall symptoms (16). Conversely, rTMS may not be effective in patients with severe TBI (19) and if it is applied years after mild to moderate TBI (avg. 8.4 years) (20). These rTMS data suggest that rTMS may be effective in modulating post-TBI cellular activity, and the effectiveness of rTMS may be influenced by the timing of administration and severity of the initial injury. Nonetheless, all RCTs in this domain are underpowered (n = 9 to 30) to inform a protocol used in clinical settings.

Several therapeutic interventions, such as blue light therapy, acupressure, and acupuncture that can impact the connectivity of the neurosenal network, have yielded beneficial effects. Following 30-min blue light therapy sessions for 6 weeks, patients with mTBI greatly benefited in the context of their cognitive functioning, daytime sleepiness, and fatigue (44, 45). Exposure to blue light, as opposed to amber light, has a positive impact on gray matter volume in the posterior thalamus and greater structural and functional connectivity in the prefrontal cortex and thalamus, which has been shown to be associated with improvements in both cognitive performance and sleep/fatigue (44, 62, 63). Likewise, both acupressure (64) and acupuncture (65) reported significant improvements in cognitive function in patients with mild to moderate TBI, which was partly attributed to the modulation in the limbic-paralimbic neocortical network and subcortical gray matter (65), along with an increased relaxation response (64, 65). Taken together, non-invasive neurostimulation interventions have the potency to facilitate healthy brain network connections, and researchers and clinicians should be encouraged to conduct a larger-scale RCT in patients with chronic TBI symptoms.

### 4.3 Exercise-based intervention

Exercise-based interventions have been spotlighted in TBI care, owing to the pioneer works (BCTT) done by John Leddy et al. (66). Patients with PPCS often report lingering symptoms including depression, anxiety, and decreased quality of life (49). Despite the RCTs in this review focused on patients with a history of moderate to severe TBI, these RCT results replicated ones from newer RCTs focusing on sports-related mTBI and pediatric mTBI. More specifically, light to moderate exercise or walking, when it is frequent (e.g., 1 h for 3 times a week for 6 to 8 weeks), resulted in improvements in mood, stress, and overall quality of life (48, 49, 67), whereas the shorter duration of exercise (e.g., 30-min per session) and self-guided walking without detailed instruction did not improve any TBI symptoms. This dose and intensity-dependent results may suggest that there is a threshold for inducing positive effects. Exercise interventions during recovery from TBI are aimed not only to increase systemic and cerebral blood flows and regulation of autonomic nerve function, but also to improve cortical connectivity and activation and overexpress brain-derived neurotrophic factor (BDNF). While it is not recommended to engage in high-intensity activity to exacerbate TBI symptoms, reaching certain physiological thresholds may be a key to designing an effective treatment protocol for patients. Although self-guided movement activity, such as Tai Chi, does not induce the same physiological reaction as walking or running, two RCTs using Tai Chi have shown consistent improvements in patients’ mood (46, 47), which is likely through the breathing technique inducing participants to relax and relieve tensions.

### 4.4 Publication bias

It is important to acknowledge the potential publication bias, often encountered in systematic reviews. This bias occurs when studies with positive or significant findings are more likely to be published, whereas negative or non-significant results may face difficulty in publication. This bias is particularly relevant for smaller-scale pilot RCTs using stimulation- and exercise-based interventions because double-blinding is often infeasible and relative easiness of conducting a pilot RCT due to cost-friendly and less logistical hurdles compared to pharmacological-based RCT, which involves additional layers of regulatory clearance (e.g., FDA). As a result, higher risk of bias was noted especially in the exercise-based interventions (Supplementary Table 1).

### 4.5 Limitations

While there were several promising candidate interventions, some limitations should be noted to propel future clinical trials. Many, but not all, RCTs had small sample sizes of less than 30 patients with TBI. This trend was prominent in relatively newer interventions (e.g., rTMS, Tai Chi, virtual reality); thus, data from underpowered pilot RCTs should be interpreted with caution. Unlike interventions for acute TBI, research focusing on chronic TBI includes a wide range of patients in terms of time since injury (e.g., 1 month to several years). This introduces other confounding factors, such as lifestyle, occupation, and developmental stages after TBI. Regardless of these potential limitations, some interventions were able to elicit beneficial effects on cognition, mood/mental health, and sleep/fatigue. Initial severities of TBI often determine the procedures for acute triage and care. However, severities of TBI begin to intersect after several months to years, with an example of some moderate TBI patients can recover quickly (68), whereas (68), whereas mild TBI does not mean patients recover patients recover rapidly, in fact some patients can develop lingering symptoms, whereas mild TBI does not mean patients recover rapidly, in fact some patients can develop lingering symptoms for months to years (69). Therefore, initial severities of TBI in the selected RCTs may be less relevant when interpreting the effectiveness of interventions for chronic TBI cases.

### 4.6 Future directions

TBI is a complex condition that can lead to persistent chronic symptoms lasting for months to even years. Given the high prevalence rates of TBI and its potential implications for later-onset neurodegenerative conditions, there is a crucial need for continuous exploration of effective interventions. Research into candidate pharmaceutical interventions, including neurostimulant medications and growth hormone, would significantly benefit from a more rigorous study design. This entails implementing a more uniform inclusion timeline (e.g., within 3–6 months post-TBI), conducting multi-center trials, and extending follow-up time points. Moreover, gaining a deeper mechanistic understanding through preclinical investigations of these medications can provide valuable insights for the clinical care of TBI patients. Brain stimulation interventions, particularly rTMS and blue-light therapy, necessitate larger sample sizes to replicate previous research findings. This approach aims to determine optimal dosage, intensity, and duration that effectively expedite the recovery process. On a parallel note, exercise-based interventions have gained traction across various health domains, including concussion and TBI care. Despite the relative ease and cost-friendliness of implementing such interventions, there is a need for improved research rigor. Standardizing sessions, dosage, and duration of exercise, along with incorporating physiological parameters to monitor the extent of physiological load, would significantly enhance our understanding of the mechanistic link between exercise intervention and improvements in cognitive and physical symptoms after TBI. While this systematic review focuses on the effects of pharmacological, stimulation, and exercise-based interventions on the chronic phases of TBI, it remains imperative to compare and contrast their effectiveness between acute and chronic phases. This consideration is especially crucial in light of a recent systematic review pointing to the pharmacological efficacy in acute TBI (70).

## 5 Conclusion

This systematic review revealed several key trends. Several hormone-based (growth hormone, melatonin) and anti-inflammatory/oxidant (Enzogenol, MLC901) medications were associated with robust improvements in executive function, but not mental state or overall TBI symptoms. A series of neurotransmitter-modulation medicines and neurostimulants had inconsistent effects on outcomes, regardless of initial TBI severity. rTMS appears to be a beneficial therapy but its effectiveness is influenced by the timing of administration and severity of the initial injury; the milder the TBI severity and the quicker TMS is delivered, the higher the likelihood of a beneficial outcome. However, given the small sample sizes, a large-scale RCT using the TMS technique is needed. Despite being in the early stages of the investigation, blue light therapy, acupressure, and acupuncture show promise for improved outcomes in chronic TBI patients, whereas tDCS does not seem to have a benefit in this population. Various independent exercise protocols have shown potent effects in improving mood, stress, and overall mental health well-being, encouraging follow-up larger-scale RCTs to confirm previous data from pilot RCTs.

## Data availability statement

The original contributions presented in the study are included in the article/Supplementary material, further inquiries can be directed to the corresponding author.

## Author contributions

KK: Writing – review & editing, Writing – original draft, Visualization, Supervision, Investigation, Funding acquisition, Conceptualization. DR: Writing – review & editing, Writing – original draft, Methodology, Investigation, Formal analysis, Data curation. CT: Writing – review & editing, Writing – original draft, Methodology, Formal analysis, Data curation, Conceptualization. RM: Writing – review & editing, Methodology, Investigation, Conceptualization. JB: Writing – review & editing, Methodology, Investigation, Conceptualization. DD: Writing – review & editing, Supervision, Methodology, Investigation, Conceptualization.

## References

[1] Hyder AA Wunderlich CA Puvanachandra P Gururaj G Kobusingye OC. The impact of traumatic brain injuries: a global perspective. NeuroRehabilitation. (2007) 22:341–53. doi: 10.3233/NRE-2007-22502, PMID: 18162698

[2] Haarbauer-Krupa J Pugh MJ Prager EM Harmon N Wolfe J Yaffe K. Epidemiology of chronic effects of traumatic brain injury. J Neurotrauma. (2021) 38:3235–47. doi: 10.1089/neu.2021.0062, PMID: 33947273 PMC9122127

[3] Howell DR Zemek R Brilliant AN Mannix RC Master CL Meehan WP 3rd. Identifying persistent Postconcussion symptom risk in a Pediatric sports medicine clinic. Am J Sports Med. (2018) 46:3254–61. doi: 10.1177/0363546518796830, PMID: 30265817

[4] Novak Z Aglipay M Barrowman N Yeates KO Beauchamp MH Gravel J . Emergency research Canada predicting persistent Postconcussive problems in pediatrics concussion, Association of Persistent Postconcussion Symptoms with Pediatric Quality of life. JAMA Pediatr. (2016) 170:e162900. doi: 10.1001/jamapediatrics.2016.2900, PMID: 27775762

[5] Carney N Totten AM O'Reilly C Ullman JS Hawryluk GW Bell MJ . Guidelines for the Management of Severe Traumatic Brain Injury, fourth edition. Neurosurgery. (2017) 80:6–15. doi: 10.1227/NEU.0000000000001432, PMID: 27654000

[6] McCrory P Meeuwisse W Dvorak J Aubry M Bailes J Broglio S . Consensus statement on concussion in sport-the 5(th) international conference on concussion in sport held in Berlin. Br J Sports Med. (2016) 51:838–47. doi: 10.1136/bjsports-2017-097699 28446457

[7] Dijkers MP . Quality of life after traumatic brain injury: a review of research approaches and findings. Arch Phys Med Rehabil. (2004) 85:S21–35. doi: 10.1016/j.apmr.2003.08.119, PMID: 15083419

[8] Vasterling JJ Jacob SN Rasmusson A. Traumatic brain injury and posttraumatic stress disorder: conceptual, diagnostic, and therapeutic considerations in the context of co-occurrence. J Neuropsychiatry Clin Neurosci. (2018) 30:91–100. doi: 10.1176/appi.neuropsych.17090180, PMID: 29132272

[9] Schwarzbold M Diaz A Martins ET Rufino A Amante LN Thais ME . Psychiatric disorders and traumatic brain injury. Neuropsychiatr Dis Treat. (2008) 4:797–816. doi: 10.2147/ndt.s2653, PMID: 19043523 PMC2536546

[10] Johansson B Wentzel AP Andrell P Odenstedt J Mannheimer C Ronnback L. Evaluation of dosage, safety and effects of methylphenidate on post-traumatic brain injury symptoms with a focus on mental fatigue and pain. Brain Inj. (2014) 28:304–10. doi: 10.3109/02699052.2013.865267, PMID: 24377326

[11] Kaiser PR Valko PO Werth E Thomann J Meier J Stocker R . Modafinil ameliorates excessive daytime sleepiness after traumatic brain injury. Neurology. (2010) 75:1780–5. doi: 10.1212/WNL.0b013e3181fd62a2, PMID: 21079179

[12] Menn SJ Yang R Lankford A. Armodafinil for the treatment of excessive sleepiness associated with mild or moderate closed traumatic brain injury: a 12-week, randomized, double-blind study followed by a 12-month open-label extension. J Clin Sleep Med. (2014) 10:1181–91. doi: 10.5664/jcsm.4196, PMID: 25325609 PMC4224718

[13] Berginstrom N Nordstrom P Schuit R Nordstrom A. The effects of (−)-OSU6162 on chronic fatigue in patients with traumatic brain injury: a randomized controlled trial. J Head Trauma Rehabil. (2017) 32:E46–54. doi: 10.1097/HTR.0000000000000236, PMID: 27120292

[14] Mansour NO Shama MA Werida RH. The effect of doxycycline on neuron-specific enolase in patients with traumatic brain injury: a randomized controlled trial. Ther Adv Chronic Dis. (2021) 12:20406223211024362. doi: 10.1177/20406223211024362 34262678 PMC8246481

[15] Choi GS Kwak SG Lee HD Chang MC. Effect of high-frequency repetitive transcranial magnetic stimulation on chronic central pain after mild traumatic brain injury: a pilot study. J Rehabil Med. (2018) 50:246–52. doi: 10.2340/16501977-2321, PMID: 29392332

[16] Moussavi Z Suleiman A Rutherford G Ranjbar Pouya O Dastgheib Z Zhang W . A pilot randomised double-blind study of the tolerability and efficacy of repetitive transcranial magnetic stimulation on persistent post-concussion syndrome. Sci Rep. (2019) 9:5498. doi: 10.1038/s41598-019-41923-6, PMID: 30940870 PMC6445141

[17] Lee SA Kim MK. Effect of low frequency repetitive transcranial magnetic stimulation on depression and cognition of patients with traumatic brain injury: a randomized controlled trial. Med Sci Monit. (2018) 24:8789–94. doi: 10.12659/MSM.911385, PMID: 30513530 PMC6289027

[18] Hoy KE McQueen S Elliot D Herring SE Maller JJ Fitzgerald PB. A pilot investigation of repetitive transcranial magnetic stimulation for post-traumatic brain injury depression: safety, tolerability, and efficacy. J Neurotrauma. (2019) 36:2092–8. doi: 10.1089/neu.2018.6097, PMID: 30712461

[19] Neville IS Zaninotto AL Hayashi CY Rodrigues PA Galhardoni R Ciampi de Andrade D . Repetitive TMS does not improve cognition in patients with TBI: a randomized double-blind trial. Neurology. (2019) 93:e190–9. doi: 10.1212/WNL.0000000000007748, PMID: 31175209 PMC6656650

[20] Siddiqi SH Trapp NT Hacker CD Laumann TO Kandala S Hong X . Repetitive transcranial magnetic stimulation with resting-state network targeting for treatment-resistant depression in traumatic brain injury: a randomized, controlled, double-blinded pilot study. J Neurotrauma. (2019) 36:1361–74. doi: 10.1089/neu.2018.5889, PMID: 30381997 PMC6909726

[21] Leddy JJ Kozlowski K Donnelly JP Pendergast DR Epstein LH Willer B. A preliminary study of subsymptom threshold exercise training for refractory post-concussion syndrome. Clin J Sport Med. (2010) 20:21–7. doi: 10.1097/JSM.0b013e3181c6c22c, PMID: 20051730

[22] Patricios JS Schneider KJ Dvorak J Ahmed OH Blauwet C Cantu RC . Consensus statement on concussion in sport: the 6th international conference on concussion in sport-Amsterdam, October 2022. Br J Sports Med. (2023) 57:695–711. doi: 10.1136/bjsports-2023-106898, PMID: 37316210

[23] Tan CO Meehan WP 3rd Iverson GL Taylor JA. Cerebrovascular regulation, exercise, and mild traumatic brain injury. Neurology. (2014) 83:1665–72. doi: 10.1212/WNL.0000000000000944, PMID: 25274845 PMC4223082

[24] Griffin EW Mullally S Foley C Warmington SA O'Mara SM Kelly AM. Aerobic exercise improves hippocampal function and increases BDNF in the serum of young adult males. Physiol Behav. (2011) 104:934–41. doi: 10.1016/j.physbeh.2011.06.005, PMID: 21722657

[25] Stroth S Hille K Spitzer M Reinhardt R. Aerobic endurance exercise benefits memory and affect in young adults. Neuropsychol Rehabil. (2009) 19:223–43. doi: 10.1080/09602010802091183, PMID: 18609015

[26] Mikkelsen K Stojanovska L Polenakovic M Bosevski M Apostolopoulos V. Exercise and mental health. Maturitas. (2017) 106:48–56. doi: 10.1016/j.maturitas.2017.09.003 29150166

[27] Leddy JJ Master CL Mannix R Wiebe DJ Grady MF Meehan WP . Early targeted heart rate aerobic exercise versus placebo stretching for sport-related concussion in adolescents: a randomised controlled trial. Lancet Child Adolesc Health. (2021) 5:792–9. doi: 10.1016/S2352-4642(21)00267-4, PMID: 34600629

[28] Wingerson MJ Hunt DL Wilson JC Mannix RC Meehan WP Howell DR. Factors associated with symptom resolution after aerobic exercise intervention in adolescent and young adults with concussion. Med Sci Sports Exerc. (2023). doi: 10.1249/MSS.0000000000003358, PMID: 38109187 PMC11018463

[29] Moher D Liberati A Tetzlaff J Altman DG and the PRISMA Group. Reprint—preferred reporting items for systematic reviews and Meta-analyses: the PRISMA statement. Phys Ther. (2009) 89:873–80. doi: 10.1093/ptj/89.9.873, PMID: 19723669

[30] Alashram AR Annino G Raju M Padua E. Effects of physical therapy interventions on balance ability in people with traumatic brain injury: a systematic review. NeuroRehabilitation. (2020) 46:455–66. doi: 10.3233/NRE-203047 32508337

[31] Schlemmer E Nicholson N. Vestibular rehabilitation effectiveness for adults with mild traumatic brain injury/concussion: a Mini-systematic review. Am J Audiol. (2022) 31:228–42. doi: 10.1044/2021_AJA-21-00165, PMID: 35077655

[32] Schneider KJ Leddy JJ Guskiewicz KM Seifert T McCrea M Silverberg ND . Rest and treatment/rehabilitation following sport-related concussion: a systematic review. Br J Sports Med. (2017) 51:930–4. doi: 10.1136/bjsports-2016-097475, PMID: 28341726

[33] Higgins JP Savovic J Page MJ Elbers RG Sterne JAC. Chapter 8: assessing risk of bias in a randomized trial In: Cochrane handbook for systematic reviews of interventions (2008)

[34] Higgins JPT Savović J Page MJ Elbers RG Sterne JAC. “Chapter 8: Assessing risk of bias in a randomized trial,” in Cochrane Handbook for Systematic Reviews of Interventions version 6.4. Eds. Higgins JPT, Thomas J, Chandler J, Cumpston M, Li T, Page MJ, Welch VA. Cochrane (2023).

[35] Lequerica A Jasey N Portelli Tremont JN Chiaravalloti ND. Pilot study on the effect of Ramelteon on sleep disturbance after traumatic brain injury: preliminary evidence from a clinical trial. Arch Phys Med Rehabil. (2015) 96:1802–9. doi: 10.1016/j.apmr.2015.05.011, PMID: 26026580

[36] Theadom A Barker-Collo S Jones KM Parmar P Bhattacharjee R Feigin VL. MLC901 (NeuroAiD II) for cognition after traumatic brain injury: a pilot randomized clinical trial. Eur J Neurol. (2018) 25:1055–e82. doi: 10.1111/ene.13653, PMID: 29611892 PMC6055867

[37] Kim YH Ko MH Na SY Park SH Kim KW. Effects of single-dose methylphenidate on cognitive performance in patients with traumatic brain injury: a double-blind placebo-controlled study. Clin Rehabil. (2006) 20:24–30. doi: 10.1191/0269215506cr927oa, PMID: 16502746

[38] Dorer CL Manktelow AE Allanson J Sahakian BJ Pickard JD Bateman A . Methylphenidate-mediated motor control network enhancement in patients with traumatic brain injury. Brain Inj. (2018) 32:1040–9. doi: 10.1080/02699052.2018.1469166, PMID: 29738277

[39] Hart T Whyte J Watanabe T Chervoneva I. Effects of dextroamphetamine in subacute traumatic brain injury: a randomized, placebo-controlled pilot study. J Neurosci Res. (2018) 96:702–10. doi: 10.1002/jnr.24102, PMID: 28653428

[40] Ripley DL Morey CE Gerber D Harrison-Felix C Brenner LA Pretz CR . Atomoxetine for attention deficits following traumatic brain injury: results from a randomized controlled trial. Brain Inj. (2014) 28:1514–22. doi: 10.3109/02699052.2014.919530 25180876

[41] Silver JM Koumaras B Chen M Mirski D Potkin SG Reyes P . Effects of rivastigmine on cognitive function in patients with traumatic brain injury. Neurology. (2006) 67:748–55. doi: 10.1212/01.wnl.0000234062.98062.e9, PMID: 16966534

[42] Kang EK Kim DY Paik NJ. Transcranial direct current stimulation of the left prefrontal cortex improves attention in patients with traumatic brain injury: a pilot study. J Rehabil Med. (2012) 44:346–50. doi: 10.2340/16501977-0947, PMID: 22434324

[43] Lesniak M Polanowska K Seniow J Czlonkowska A. Effects of repeated anodal tDCS coupled with cognitive training for patients with severe traumatic brain injury: a pilot randomized controlled trial. J Head Trauma Rehabil. (2014) 29:E20–9. doi: 10.1097/HTR.0b013e318292a4c2, PMID: 23756431

[44] Killgore WDS Vanuk JR Shane BR Weber M Bajaj S. A randomized, double-blind, placebo-controlled trial of blue wavelength light exposure on sleep and recovery of brain structure, function, and cognition following mild traumatic brain injury. Neurobiol Dis. (2020) 134:104679. doi: 10.1016/j.nbd.2019.104679, PMID: 31751607

[45] Sinclair KL Ponsford JL Taffe J Lockley SW Rajaratnam SM. Randomized controlled trial of light therapy for fatigue following traumatic brain injury. Neurorehabil Neural Repair. (2014) 28:303–13. doi: 10.1177/1545968313508472, PMID: 24213962

[46] Blake H Batson M. Exercise intervention in brain injury: a pilot randomized study of tai chi qigong. Clin Rehabil. (2009) 23:589–98. doi: 10.1177/0269215508101736, PMID: 19237436

[47] Gemmell C Leathem JM. A study investigating the effects of tai chi Chuan: individuals with traumatic brain injury compared to controls. Brain Inj. (2006) 20:151–6. doi: 10.1080/02699050500442998, PMID: 16421063

[48] Driver S Ede A. Impact of physical activity on mood after TBI. Brain Inj. (2009) 23:203–12. doi: 10.1080/02699050802695574, PMID: 19205956

[49] Wise EK Hoffman JM Powell JM Bombardier CH Bell KR. Benefits of exercise maintenance after traumatic brain injury. Arch Phys Med Rehabil. (2012) 93:1319–23. doi: 10.1016/j.apmr.2012.05.009, PMID: 22840829

[50] Jacoby M Averbuch S Sacher Y Katz N Weiss PL Kizony R. Effectiveness of executive functions training within a virtual supermarket for adults with traumatic brain injury: a pilot study. IEEE Trans Neural Syst Rehabil Eng. (2013) 21:182–90. doi: 10.1109/TNSRE.2012.2235184 23292820

[51] Tefertiller C Hays K Natale A O'Dell D Ketchum J Sevigny M . Results from a randomized controlled trial to address balance deficits after traumatic brain injury. Arch Phys Med Rehabil. (2019) 100:1409–16. doi: 10.1016/j.apmr.2019.03.015, PMID: 31009598 PMC8594144

[52] Jensen OK Nielsen FF Vosmar L. An open study comparing manual therapy with the use of cold packs in the treatment of post-traumatic headache. Cephalalgia. (1990) 10:241–50. doi: 10.1046/j.1468-2982.1990.1005241.x, PMID: 2272094

[53] Chizuk HM Willer BS Cunningham A Bezherano I Storey E Master C . Adolescents with sport-related concussion who adhere to aerobic exercise prescriptions recover faster. Med Sci Sports Exerc. (2022) 54:1410–6. doi: 10.1249/MSS.0000000000002952, PMID: 35482774 PMC9378725

[54] Micay R Richards D Hutchison MG. Feasibility of a postacute structured aerobic exercise intervention following sport concussion in symptomatic adolescents: a randomised controlled study. BMJ Open Sport Exerc Med. (2018) 4:e000404. doi: 10.1136/bmjsem-2018-000404, PMID: 30018795 PMC6045733

[55] Leddy JJ Haider MN Ellis MJ Mannix R Darling SR Freitas MS . Early subthreshold aerobic exercise for sport-related concussion: a randomized clinical trial. JAMA Pediatr. (2019) 173:319–25. doi: 10.1001/jamapediatrics.2018.4397, PMID: 30715132 PMC6450274

[56] Theadom A Mahon S Barker-Collo S McPherson K Rush E Vandal AC . Enzogenol for cognitive functioning in traumatic brain injury: a pilot placebo-controlled RCT. Eur J Neurol. (2013) 20:1135–44. doi: 10.1111/ene.12099, PMID: 23384428

[57] Widmann C Gandin C Petit-Paitel A Lazdunski M Heurteaux C. The traditional Chinese medicine MLC901 inhibits inflammation processes after focal cerebral ischemia. Sci Rep. (2018) 8:18062. doi: 10.1038/s41598-018-36138-0, PMID: 30584250 PMC6305383

[58] Quintard H Lorivel T Gandin C Lazdunski M Heurteaux C. MLC901, a traditional Chinese medicine induces neuroprotective and neuroregenerative benefits after traumatic brain injury in rats. Neuroscience. (2014) 277:72–86. doi: 10.1016/j.neuroscience.2014.06.047, PMID: 24993477

[59] Di Pietro V Yakoub KM Caruso G Lazzarino G Signoretti S Barbey AK . Antioxidant therapies in traumatic brain injury. Antioxidants. (2020) 9:260. doi: 10.3390/antiox9030260 32235799 PMC7139349

[60] Liu J Clough SJ Hutchinson AJ Adamah-Biassi EB Popovska-Gorevski M Dubocovich ML. MT1 and MT2 melatonin receptors: a therapeutic perspective. Annu Rev Pharmacol Toxicol. (2016) 56:361–83. doi: 10.1146/annurev-pharmtox-010814-124742, PMID: 26514204 PMC5091650

[61] Eldaief MC Press DZ Pascual-Leone A. Transcranial magnetic stimulation in neurology: a review of established and prospective applications. Neurol Clin Pract. (2013) 3:519–26. doi: 10.1212/01.CPJ.0000436213.11132.8e, PMID: 24353923 PMC3863979

[62] Bajaj S Raikes AC Razi A Miller MA Killgore WD. Blue-light therapy strengthens resting-state effective connectivity within default-mode network after mild TBI. J Cent Nerv Syst Dis. (2021) 13:11795735211015076. doi: 10.1177/11795735211015076 34104033 PMC8145607

[63] Raikes AC Dailey NS Forbeck B Alkozei A Killgore WDS. Daily morning blue light therapy for post-mTBI sleep disruption: effects on brain structure and function. Front Neurol. (2021) 12:625431. doi: 10.3389/fneur.2021.625431, PMID: 33633674 PMC7901882

[64] McFadden KL Healy KM Dettmann ML Kaye JT Ito TA Hernandez TD. Acupressure as a non-pharmacological intervention for traumatic brain injury (TBI). J Neurotrauma. (2011) 28:21–34. doi: 10.1089/neu.2010.1515, PMID: 20979460

[65] Zollman FS Larson EB Wasek-Throm LK Cyborski CM Bode RK. Acupuncture for treatment of insomnia in patients with traumatic brain injury: a pilot intervention study. J Head Trauma Rehabil. (2012) 27:135–42. doi: 10.1097/HTR.0b013e3182051397 21386714

[66] Leddy JJ Willer B. Use of graded exercise testing in concussion and return-to-activity management. Curr Sports Med Rep. (2013) 12:370–6. doi: 10.1249/JSR.0000000000000008, PMID: 24225521

[67] Bellon K Kolakowsky-Hayner S Wright J Huie H Toda K Bushnik T . A home-based walking study to ameliorate perceived stress and depressive symptoms in people with a traumatic brain injury. Brain Inj. (2015) 29:313–9. doi: 10.3109/02699052.2014.974670, PMID: 25356799

[68] Nelson LD Temkin NR Barber J Brett BL Okonkwo DO McCrea MA . Functional recovery, symptoms, and quality of life 1 to 5 years after traumatic brain injury. JAMA Netw Open. (2023) 6:e233660. doi: 10.1001/jamanetworkopen.2023.3660, PMID: 36939699 PMC10028488

[69] Rabinowitz AR Li X McCauley SR Wilde EA Barnes A Hanten G . Prevalence and predictors of poor recovery from mild traumatic brain injury. J Neurotrauma. (2015) 32:1488–96. doi: 10.1089/neu.2014.3555, PMID: 25970233 PMC4702434

[70] Mansour NO Elnaem MH Abdelaziz DH Barakat M Dehele IS Elrggal ME . Effects of early adjunctive pharmacotherapy on serum levels of brain injury biomarkers in patients with traumatic brain injury: a systematic review of randomized controlled studies. Front Pharmacol. (2023) 14:1185277. doi: 10.3389/fphar.2023.1185277, PMID: 37214454 PMC10196026

