# Supplementary material for: Effectiveness of biomedical interventions on the chronic stage of traumatic brain injury: a systematic review of randomized controlled trials
Source: Front Neurol. 2024 Mar 18;15:1321239. doi: 10.3389/fneur.2024.1321239 (PMC10983769; doi:10.3389/fneur.2024.1321239)
Supplement: Supplementary file 1 [file Table_1.DOCX]

| Supplemental Table 1: Bias of the included studies.  (+/-) unclear risk of bias; (-) high risk of bias; (+) low risk of bias | **Sequence generation – Selection bias** | **Allocation sequence concealment – Selection bias** | **Blinding of participants and personnel – Performance bias** | **Blinding of outcome assessment – Detection bias** | **Incomplete outcome data** | **Selective outcome reporting – Reporting bias** | **Other bias** |
| --- | --- | --- | --- | --- | --- | --- | --- |
| **Pharmacological Interventions** | | | | | | | |
| **Tenovuo et al. 2004** | - | - | - | - | + | + | - |
| **Zhang et al. 2004** | + | + | + | + | + | + | + |
| **Kim et al. 2006** | + | + | + | + | + | + | + |
| **Silver et al. 2006** | + | +/- | + | + | + | + | + |
| **Amitabh et al. 2008** | - | - | + | +/- | + | + | + |
| **High et al. 2010** | + | - | + | + | + | + | + |
| **Kaiser et al. 2010** | + | + | + | + | + | + | + |
| **Giacino et al. 2012** | - | - | + | + | + | +/- | + |
| **Johansson et al. 2013** | +/- | +/- | + | + | + | + | + |
| **Theadom et al. 2013** | + | +/- | + | + | + | + | + |
| **Menn et al. 2014** | - | - | + | + | + | + | + |
| **Ripley et al. 2014** | + | + | + | + | + | + | + |
| **Hammond et al. 2015** | + | + | + | + | + | + | + |
| **Lequerica et al. 2015** | + | + | + | + | + | + | + |
| **Berginstrom et al. 2017** | + | + | + | + | + | + | + |
| **Hart et al. 2017** | - | - | +/- | +/- | + | + | + |
| **Dorer et al. 2018** | +/- | - | + | +/- | + | +/- | +/- |
| **Theadom et al. 2018** | + | + | + | + | + | + | + |
|  |  |  |  |  |  |  |  |
| **Stimulation Interventions** | | | | | | | |
| **Schoenberger et al. 2001** | +/- | - | + | +/- | + | + | + |
| **McFadden et al. 2011** | + | + | +/- | - | + | + | + |
| **Kang et al. 2012** | - | - | +/- | +/- | + | + | +/- |
| **Zollman et al. 2012** | - | - | - | - | + | + | - |
| **Lesniak et al. 2014** | +/- | - | + | + | + | + | + |
| **Sinclair et al. 2014** | + | + | + | + | + | + | + |
| **Choi et al. 2018** | + | +/- | + | +/- | + | + | + |
| **Lee et al. 2018** | + | + | + | + | + | + | + |
| **Hoy et al. 2019** | + | +/- | + | + | + | + | + |
| **Moussavi et al. 2019** | + | +/- | + | + | + | + | + |
| **Neville et al. 2019** | + | + | + | + | + | + | + |
| **Siddiqi et al. 2019** | - | - | + | +/- | + | + | +/- |
| **Zhang et al. 2019** | + | + | + | + | + | + | + |
| **Killogore et al. 2020** | - | - | +/- | - | + | - | - |
|  |  |  |  |  |  |  |  |
| **Exercise-based Interventions** | | | | | | | |
| **Jensen et al. 1990** | + | + | +/- | +/- | + | + | + |
| **Gemmell et al. 2006** | - | - | - | +/- | +/- | +/- | - |
| **Wilson et al. 2006** | + | + | + | + | + | + | + |
| **Blake et al. 2009** | + | +/- | + | + | + | + | + |
| **Driver et al. 2009** | - | - | - | - | +/- | +/- | - |
| **Hoffman et al. 2010** | + | + | +/- | +/- | + | + | + |
| **Wise et al. 2012** | - | - | +/- | - | + | +/- | - |
| **Jacoby et al. 2013** | - | - | +/- | +/- | +/- | + | - |
| **Bellon et al. 2014** | + | +/- | + | + | + | + | + |
| **Kalokowsky-Hayner et al. 2016** | + | - | - | +/- | + | + | + |
| **Chiu et al. 2017** | + | + | + | + | + | + | + |
| **Tefertiller et al. 2019** | - | - | +/- | + | + | + | + |
|  |  |  |  |  |  |  |  |

(+/-) unclear risk of bias; (-) high risk of bias; (+) low risk of bias
